# Intimate partner violence against women during covid-19: A population-based study in Vitória, state of Espírito Santo, Brazil

**DOI:** 10.1371/journal.pone.0295340

**Published:** 2023-12-20

**Authors:** Franciéle Marabotti Costa Leite, Bruna Venturin, Luiza Eduarda Portes Ribeiro, Ranielle De Paula Silva, Mayara Luis Alves, Fernando César Wehrmeister, Dherik Fraga Santos

**Affiliations:** 1 Postgraduate Program in Collective Health, Federal University of Espírito Santo, Vitória, Espírito Santo, Brazil; 2 Postgraduate Program in Epidemiology, Federal University of Pelotas, Pelotas, Rio Grande do Sul, Brazil; 3 Department of Medicine, Federal University of Catalão, Catalão, Goiás, Brazil; Faculdade Sao Leopoldo Mandic, BRAZIL

## Abstract

**Background:**

Violence against women has a negative impact on multiple dimensions of women’s health. During the Covid-19 pandemic, intimate partner violence against women has continued, and in some contexts has intensified. The aim of this study was to identify the prevalence of intimate partner violence against women during covid-19 pandemic and its association with socioeconomic, behavioral, and life-experience factors.

**Methods and findings:**

Cross-sectional, population-based study conducted in the municipality of Vitória, state of Espírito Santo, from January to May 2022, where 1,086 women aged 18 years and over were interviewed. The World Health Organization (WHO) instrument on violence against women was used to screen outcomes. The prevalence of violence during the pandemic (psychological, physical, and sexual) and bivariate analysis with sociodemographic, behavioral, family, and life history characteristics of women were estimated. The multivariate analysis was carried out for each type of violence, the Poisson regression model was performed with an estimate of robust variance, inserting the variables of interest with (p<0.20). Those with p<0.05 remained in the adjusted model.

**Results:**

The prevalence of violence psychological against women perpetrated by an intimate partner during the pandemic was the most frequent (20.2%), followed by physical (9.0%) and sexual violence (6.5%). Women with less schooling and who were single had a higher prevalence of physical and psychological violence, as did those with a history of sexual abuse in childhood and whose mothers had been beaten by their intimate partners. Sexual violence was more prevalent among non-white, with up to eight years of schooling, whose mothers had a history of intimate partner violence, and who consumed alcohol during four days or more (p<0.01).

**Conclusion:**

Psychological, physical, and sexual violence perpetrated by the intimate partner during the pandemic presented high magnitude among women living in Vitória. Sociodemographic, behavioral factors, and personal and maternal experiences of violence were associated with the phenomenon.

## Introduction

The SARS-CoV 2 pandemic has caused governments worldwide to issue public health emergency responses to reduce the virus’ spread and burden of health services [1]. Measures such as social distancing and interruption of classes and face-to-face work were adopted, which directly affected how individuals and families lived, since it caused isolation, loneliness, unemployment, and the risk of mental disorders, possibly leading to stress, tension, and violence [2].

The United Nations predicted a 20% increase in cases of violence against women during the pandemic due to lockdown and recommendations to stay at home [3]. A study with police records from Norway showed a 54% increase in reports of intimate partner violence during lockdown [4]. In Peru, telephone reports of violence against women increased 48% from April to July 2020 [5]. In Brazil, records of violence against women increased 17% in March 2020, when the first restrictive measures were enacted in the country [6].

A study conducted in Germany with 3,818 women with partners during April and May 2020 presented a 25.3% prevalence of physical violence, 3.09% of psychological violence and 3.57% of sexual abuse. Women in home isolation had twice the risk of physical violence compared to those not in isolation [7]. In Italy, a study with a sample of women treated at a reference center for violence during the pandemic showed a 2.61 times greater chance of increased frequency of violence in women who lived with their partners compared to those who did not. Likewise, women who lived with their partners had a lower chance of reporting a reduced victimization of violence during the pandemic [8].

A systematic review and meta-analysis that investigated intimate partner violence against pregnant women during the COVID-19 pandemic showed that the total of IPV was estimated at 22.0%, and the prevalence of psychological, physical, and sexual violence was reported to be 24.0%, 14.0%, 6.0% respectively [9].

Partners are the main perpetrators of violence against women who experience threats, physical aggression, controlling behaviors, and jealousy within their homes, a place that should be safe, of protection and rest, but it is where violence occurs most often [10]. Moreover, violence against women is historically seen as a private matter of each family, and the home is considered an intimate area, in which the public authorities have no right to interfere [11]. In this regard, the perpetrators of intimate partner violence (IPV) took advantage on the COVID-19 restrictions adopted by many countries to increase their power and control over women, who, forced to spend time at home, may be isolated and unable to seek help [12].

The occurrence of violence is shaped by social, economic, and cultural contexts, and during health crises inequalities are increased, including those based on socioeconomic aspects, age, race, and gender [12]. Among the factors associated with IPV already recognized in the literature such as the partner’s low level of education or income, the woman’s marital status, religion, low social support, alcohol consumption, history of violence throughout life and dominating behaviours by the partner [7, 8]. Besides, the pandemic is associated with factors that can intensify violence against women, such as forced cohabitation with their partners, difficulty in obtaining help, increased alcohol consumption, the fragility of social and professional support networks, and the stress related to economic insecurity and the fear of losing their jobs [6, 13, 14].

The study on the subject still requires a lot of progress so that government agencies can establish more effective protection policies, especially in Brazil, which still has a low representation of specific research on violence against women during the pandemic period when compared to other countries [9, 15, 16].

Therefore, knowing the magnitude, pattern of occurrence, and factors associated with violence against women during the pandemic, especially by population-based data, is essential for coping. In this context, this study aimed to identify the prevalence of violence against women during the COVID-19 pandemic and its association with socioeconomic, behavioral, and life experience factors.

## Methods

### Study design

This is a cross-sectional analytical population-based study conducted in the municipality of Vitória, capital of the state of Espírito Santo, Brazil.

### Setting

Vitória is a city in the southeastern region of Brazil with a population of 322,869 inhabitants and a demographic density of 3,324 inhabitants per km2 [17]. In 2021 according to estimates, the municipality had 196,018 women, 83% of which were women aged 15 years or older [18]. Since the identification of the first case in the state of Espírito Santo, January 2022 and February were the months with the highest number of cases identified, registering almost 273 thousand and 123 thousand confirmed cases, respectively. The municipality of Vitória follows the same pattern, registering the highest number of cases in January with almost 30 thousand cases [19]. As sanitary containment measures, elective services were suspended in order to reduce the flow of people and the chain of transmission within health services [20].

### Participants

The municipality has 79 neighborhoods and six health regions [21, 22]. According to data from the last demographic census, the female population was about 163,853 (49.98%).

The study population consisted of women aged 18 years or over, who had or have had an intimate partner in the 24 months prior to the interview. This study defined the intimate partner as the current or former partner, provided that they have sexual relations, irrespective if they are in a formal union. Women who could not understand or communicate due to intellectual or sensory deficit and thus, unable to respond to the research data collection instruments, were excluded.

### Procedures

From January to May 2022, data were collected by a team of properly trained female interviewers. The team also had field supervisors. The pilot study was conducted in December 2021. The data collected in the pilot study were not part of the study’s final sample. Fieldwork began after data analysis of the pilot study. The interviews were conducted in the women’s households, in a reserved place, respecting their privacy and confidentiality. Data were collected using tablets where the questionnaires were inserted using the redcap application. Prior to their participation in the study, the participants agreed and signed the informed consent form.

### Data sources / measurement

The study outcomes were the three types of violence against women (psychological, sexual, or physical) perpetrated by the intimate partner in the previous 24 months, that is, during the COVID-19 pandemic in Brazil.

Violence was considered present when women answered yes to one of the items for each type of violence (psychological, physical, or sexual) identified using the World Health Organization Violence Against Women (WHO VAW STUDY) instrument translated into and validated to Brazilian Portuguese [23]. In the consistency analysis the researchers found a result for Cronbach’s alpha of 0.88 for São Paulo and 0.89 for Zona da Mata, Recife [23].

### Variables

The independent variables studied were sociodemographic characteristics, family and life experiences about violence, and behavioral characteristics. Of which:

I The sociodemographic characteristics of women were:
  a) age group (18–29; 30–39; 40; 40–49; 50–59; ≥60);
  b) self-declared skin color (white; non-white);
  c) complete years of schooling (0–8; 9–11; ≥12);
  d) family income in tertiles (1^st^: poorest; 2^nd^; 3^rd^: richest);
  e) marital status (married; consensual union; single; divorced);
  f) catholic (no or yes); evangelical (no or yes).
II Regarding family and life experiences about violence, women were asked the following questions:
  g) maternal aggression by intimate partner (yes; no);
  h) sexual violence in childhood (yes; no).
III Regarding behavioral variables, the following aspects were analyzed:
  i) average frequency of alcohol use (never; monthly or less; 2–4 times per month; 2–3 times per week; and ≥4 times per week)
  j) smoking (never smoked; current smoker; former smoker).

The main reason for not using all categories of religion is based on the fact that the two religions are the most frequent in the country, with other categories of the variable (such as religion of African origin or no religion) being less prevalent, violating some statistical assumptions when choosing to use the variable with all the religions asked, that is, with the inclusion of this variable with more categories, the model had a low quality of fit. Another factor that explains the separation between Catholic and evangelical is based on the carrying out of other studies in the same municipality where the authors chose to use this method of approach due to the better interpretability of the results according to religion, which the results point in different directions for the occurrence of types of violence according to religion [24].

### Study size and bias

A multi-stage sampling process adopted was. The primary sampling unit was the census tracts of the municipality of Vitória provided by the 2010 Census conducted by the Brazilian Institute of Geography and Statistics (IBGE) [25]. The total number of households in the urban area of Vitória, Espírito Santo, in 2010 (108,515) was divided by 100 (number of sectors to be visited) to obtain the systematic gap (1,085), respecting the probability proportional to the number of households and women within each sector. Then, the list was ordered by socioeconomic level and the number 513 (between 1 and 1,085) was randomly drawn using the statistical program R, corresponding to the number of the first sector defined. The selection of the other sectors (99) occurred by summing the systematic gap of the initial sector (184) and, thus, successively until the end of the listing.

After the selection of census tracts, the selection of households occurred randomly from the list available on the online platform by the IBGE [17]. In each household, a list of eligible women was made, that is, those who met the inclusion criteria of the study, after which one of the women was selected to answer the interview. For sample size estimation, the estimated prevalence of intimate partner violence was considered in the population studied of 50% to maximize the sample, with a 95% confidence level and 5pp acceptable error. To study the association of risk factors, a 95% level, 80% power, and 1:1 exposed/unexposed ratio were considered. To this value, 10% were added for losses, and 30% for confounding factors, achieving a sample size of 1,100 women.

### Statistical analysis

Descriptive analysis was performed presenting the crude and relative frequency. Fisher’s chi-square heterogeneity and exact tests were used in bivariate analysis according to assumptions. Poisson regression with robust variance was used to estimate the crude and adjusted prevalence ratios (PR) and their respective 95% confidence intervals (95%CI). For the multivariate analysis, the hierarchical model with four levels was adopted. The hierarchical model was built according to a conceptual structure based on literature, as explained by Victora and co-authors [26]. Some authors present in their articles the construction of hierarchical models for violence [24, 27, 28].

Initially, the variables associated with the outcomes studied (p<0.20) were included in the model, to consider possible confounding factors and remained in the model with p<0.05. In the modeling stage, the independent variables were included from the distal level (first) to the proximal level (fourth), following the order: sociodemographic factors; family experience; life experience, and behavioral characteristics. The backward selection method was used to exclude the variables. In the final model, a 5% significance level (p<0.05) was adopted. Statistical analyses were performed using the statistical program Stata^®^ version 15.1.

All interviewees signed the informed consent form, and the study’s objectives and its possible risks and benefits were clarified.

## Ethical aspects

The study project was approved by Research Ethics Committee Certificate of Submission for Ethical Appraisal No. 41628820.6.0000.5060. All interviewees signed the informed consent form and the study’s objectives and its possible risks and benefits were clarified. All interviewers followed health recommendations and acquired personal protective equipment for data collection. The interviews took place in a private environment and confidentiality was guaranteed, all participants received a leaflet on health aspects and the main services that can be sought in case of violence.

## Results

There was the participation of 1086 women in the study. The sample consisted of mostly women under 60 years old (76%), non-white (60%), with 12 years or more of schooling (49%), belonging to the first income tertile, married (55%) and Catholic (42%). About 22% of the women reported that their mother had been a victim of intimate partner violence throughout their lives, and almost 11% of the interviewees had suffered sexual violence as a child. With regard to behavioral characteristics, most women had never used alcohol (50.7%) and never smoked (74.0%) (Table 1).

**Table 1:** Prevalence of violence (psychological, physical and sexual) in the last 24 months, according to sociodemographic, behavioral characteristics, family and life experience of women living in the urban area of Vitória, Espírito Santo, Brazil, 2022 (N = 1,086).

Characteristics	Sample		Violence psychological			Violence physical			Violence sexual		
Sociodemographic	N	%	%	95%CI	p-value	%	95%CI	p-value	%	95%CI	p-value
**Age group (years)**					0.050			0.041			0,141
18 to 29	202	18.6	26.2	20.6–32.7		13.4	9.3–18.8		8.9	5.7–13.7	
30 to 39	204	18.8	23.0	17.8–29.3		10.3	6.8–15.3		6.4	3.7–10.7	
40 to 49	222	20.4	19.4	14.7–25.1		7.7	4.8–12.0		8.1	5.2–12.5	
50 to 59	196	18.1	17.4	12.7–23.3		9.7	6.3–15.7		6,6	3.9–11.1	
60 or more	262	24.1	16.0	12.1–21.0		5.3	3.2–8.8		3.4	1.8–6.5	
**Self-declared skin color**					0.002			0.004			0.002
White	436	40.2	15.6	12.5–19.3		6.0	4.1–8.6		3.7	2.3–5.9	
Non-white	650	59.8	23.2	20.1–26.6		11.1	8.0–13.7		8.5	6.6–10.9	
**Complete years of schooling**					<0.001			<0.001			<0.001
0 to 8	203	18.7	32.5	26.4–39.3		16.3	11.8–22.0		11.8	8.0–17.0	
9 to 11	352	32.4	23.3	19.2–28.0		10.8	8.0–14.5		7.7	5.3–11.0	
≥12	531	48.9	13.4	10.7–16.5		5.1	3.5–7.3		3.8	2.4–5.8	
**Family income in tertiles (N = 1.070)**					<0.001			<0.001			0.004
1st: poorest	387	36.2	29.7	25.4–34.5		15.3	12.0–19.2		9.8	7.2–13.2	
2^nd^	359	33.5	18.1	14.5–22.4		6.4	4.3–9.5		5.3	3.4–8.2	
3rd: richest	324	30.3	11.7	8.6–15.7		4.6	2.8–7.5		4.0	2.3–6.8	
**Marital status**					<0.001			<0.001			0.016
Married	601	55.3	16.8	14.0–20.0		6.5	4.8–8.8		5.2	3.6–7.2	
Consensual union	285	26.2	20.4	16.1–25.4		9.8	6.9–13.9		6.3	4.0–9.8	
Single	134	12.3	33.6	26.1–42.0		19.4	13.6–27.0		12.7	8,0–19,5	
Divorced	66	6.1	22.7	14.2–34.4		7.6	3.2–17.0		7.6	3,2–17,0	
**Catholic**					0.001			0.001			0.050
No	629	57.9	23.7	20.5–27.2		11.5	9.2–14.2		7.8	5.9–10.2	
Yes	457	42.1	15.3	12.3–18.9		5.7	3.9–8.2		4.8	3.1–7.2	
**Evangelical**					0.005			0.011			0.087
No	713	65.7	17.7	15.0–20.7		7.4	5.7–9.6		5.6	4.1–7.6	
Yes	373	34.4	24.9	20.8–29.6		12.1	9.1–15.8		8.3	5.9–11.6	
**Family and life experiences about violence**											
**Maternal aggression by intimate partner (N = 1.027)**					<0.001			<0.001			<0.001
No	800	77.9	17.4	14.9–20.2		7.3	5.6–9.3		4.6	3.4–6.3	
Yes	227	22.1	31.3	25.6–37.6		16.7	12.4–22.2		13.2	9.4–18.3	
**Sexual violence in childhood**					<0.001			0.003			0.010
No	971	89.4	18.6	16.3–21.2		8.1	6.6–10.0		5,9	5.6–7.5	
Yes	115	10.6	33.0	25.1–42.1		16.5	10.8–24.5		12.2	7.3–19.5	
**Behavioral variables**											
**Average frequency of alcohol use (N = 1.080)**					0.042			0.052			0.009
Never	548	50,7	21,2	17,9–24,8		9,3	7,1–12,0		6,2	4,5–8,6	
Monthly or less	250	23,2	18,0	13,7–23,3		6,8	4,3–10,7		5,6	3,3–9,2	
2–4 times per month	177	16,4	15,8	11,1–22,0		9,0	5,6–14,3		6,2	3,5–10,9	
2–3 times per week	92	8,5	25,0	17,2–34,8		10,9	5,9–19,0		8,7	4,4–16,4	
≥4 times per week	13	1,2	46,2	22,3–71,9		30,8	12,0–59,1		30,8	12,0–59,1	
**Smoking**					0.039			0.002			0.037
Never smoked	804	74.0	18.4	15.9–21.2		7.5	5.8–9.5		5.5	4.1–7.3	
Current smoker	137	12.6	27.0	20.2–35.1		16.8	11.4–24.0		11.0	6.8–17.4	
Former smoker	145	13.4	23.5	17.3–31.0		10.3	6.3–16.5		8.3	4.8–14.0	

N: absolute frequency; %: relative frequency; 95%CI: 95% confidence interval; a: Pearson’s chi-square test.

The prevalence of violence psychological against women by an intimate partner during the pandemic was the most frequent, with a 20.2% (95%CI 17.9–22.7), followed by physical violence (9.0%; 95%CI 7.5–10.9). Sexual violence during the pandemic had the lowest prevalence (6.5%; 95%CI 5.2–8.2).

Table 1 shows that psychological violence was related to all independent variables in the study (p<0.05). Physical violence in the previous 24 months was not related to the average frequency of alcohol use, whereas sexual violence was not related to age and being evangelical (p>0.05).

In the adjusted analysis, psychological violence was twice as high among women with 0–8 years of schooling (PR: 2.01; 95%CI 1.44–2.80). Women belonging to the second tertile of family income had 62% more occurrence of psychological violence compared to those of the third tertile (richest) (PR: 1.62; 95%CI 1.08–2.43). Single women had an 83% higher prevalence of psychological violence compared to married women, and catholic women had a 25% lower prevalence compared to non-catholic women (PR: 0.75; 95%CI 0.57–0.99). The history of mothers who have already been beaten by an intimate partner had a 34% increase in the prevalence of psychological violence (PR: 1.34; 95%CI 1.03–1.73). Women who suffered sexual violence during childhood had 1.51 times the prevalence of psychological abuse perpetrated by their partner (PR: 1.51; CI95% 1.11–2.05) (Table 2).

**Table 2:** Crude and adjusted analysis of the prevalence of psychological violence in the last 24 months according to sociodemographic characteristics, family and life and behavioral experiences among women living in an urban area in the municipality of Vitória, Espírito Santo, Brazil, 2022 (N = 1,086).

Characteristics	N	Crude analysis			Adjusted analysis		
		PR	95%CI	p-value	PR	95%CI	p-value
**Sociodemographic**							
**Age group (years)**				0.049			0.285
18 to 29		1.64	1.14–2.35		1.36	0.87–2.12	
30 to 39		1.44	0.99–2.09		1.40	0.92–2.13	
40 to 49		1.21	0.82–1.78		1.23	0.82–1.84	
50 to 59		1.08	0.72–1.64		0.95	0.63–1.42	
60 or more		Ref.			Ref.		
**Self-declared skin color**				0.003			0.948
White		Ref.			Ref.		
Non-white		1.49	1.15–1.93		1.01	0.77–1.33	
**Complete years of schooling**				<0.001			<0.001
0 to 8		2.43	1.81–3.26		2.01	1.44–2.80	
9 to 11		1.74	1.31–2.32		1.37	1.00–1.89	
≥12		Ref.			Ref.		
**Family income in tertiles**				<0.001			0.016
1st: poorest	387	2.53	1.81–3.55		1.15	0.77–1.71	
2^nd^	359	1.54	1.07–2.24		1.62	1.08–2.43	
3rd: richest	324	Ref.			Ref.		
**Marital status**				<0.001			0.002
Married		Ref.			Ref.		
Consensual union		1.21	0.91–1.62		1.16	0.87–1.55	
Single		2.00	1.48–2.69		1.83	1.33–2.50	
Divorced		1.35	0.84–2.18		1.29	0.79–2.10	
**Catholic**				0.001			0.040
No		Ref.			Ref.		
Yes		0.65	0.50–0.84		0.75	0.57–0.99	
**Evangelical**				0.004			0.659
No		Ref.			Ref.		
Yes		1.41	1.11–1.79		1.07	0.79–1.46	
**Family and life experiences about violence**							
**Maternal aggression by intimate partner**				<0.001			0.029
No		Ref.			Ref.		
Yes		1.80	1.41–2.30		1.34	1.03–1.73	
**Sexual violence in childhood**				<0.001			0.009
No		Ref.			Ref.		
Yes		1.77	1.32–2.37		1.51	1.11–2.05	
**Behavioral variables**							
**Average frequency of alcohol use**				0.019			0.370
Never		Ref.			Ref.		
Monthly or less		0.85	0.62–1.16		0.82	0.60–1.11	
2–4 times per month		0.75	0.51–1.09		0.75	0.50–1.13	
2–3 times per week		1.18	0.80–1.74		0.98	0.64–1.49	
≥4 times per week		2.18	1.19–4.01		1.23	0.70–2.17	
**Smoking**				0.034			0.171
Never smoked		Ref.			Ref.		
Current smoker		1.47	1.07–2.00		1.04	0.74–1.46	
Former smoker		1.27	0.92–1.77		1.39	0.98–1.95	

N: absolute frequency; 95%CI: 95% confidence interval; PR: prevalence ratio; Ref.: reference group.

Women with >8 eight years of schooling and with 9–11 years of schooling had a higher prevalence of physical violence compared to women with higher schooling: 215% and 81%, respectively. Single women were 2.8 times more likely to experience physical violence than married women (PR: 2.80; 95%CI 1.77–4.44), and those who did not declare themselves catholic were almost 40% less likely to experience physical violence (PR: 0.58; 95%CI 0.37–0.90). Women whose mothers were beaten by their intimate partners had a 78% higher prevalence of physical violence (PR: 1.78; 95%CI 1.21–2.61). Likewise, sexual violence during childhood represented a higher occurrence of victimization of physical abuse (Table 3).

**Table 3:** Crude and adjusted analysis of the prevalence of physical violence in the last 24 months according to sociodemographic characteristics, family and life and behavioral experiences among women living in an urban area in the municipality of Vitória, Espírito Santo, Brazil, 2022 (N = 1,086).

Characteristics	N	Crude analysis			Adjusted analysis		
		PR	95%CI	p-value	PR	95%CI	p-value
**Sociodemographic**							
**Age group (years)**				0.049			0.501
18 to 29		2.50	1.35–4.65		1.90	0.88–4.13	
30 to 39		1.93	1.00–3.70		1.94	0.90–4.17	
40 to 49		1.43	0.72–2.84		1.53	0.74–3.16	
50 to 59		1.81	0.93–3.53		1.69	0.85–3.37	
60 or more		Ref.			Ref.		
**Self-declared skin color**				0.005			0.384
White		Ref.			Ref.		
Non-white		1.86	1.21–2.86		1.23	0.77–1.95	
**Complete years of schooling**				<0.001			<0.001
0 to 8		3.20	1.97–5.18		3.15	1.99–5.00	
9 to 11		2.12	1.32–3.41		1.81	1.13–2.90	
≥12		Ref.			Ref.		
**Family income in tertiles**				<0.001			0.089
1st: poorest	387	3.29	1.90–5.69		0.93	0.49–1.80	
2^nd^	359	1.38	0.73–2.61		1.56	0.81–3.03	
3rd: richest	324	Ref.			Ref.		
**Marital status**				<0.001			0.002
Married		Ref.			Ref.		
Consensual union		1.51	0.95–2.41		1.49	0.94–2.35	
Single		2.99	1.89–4.73		2.80	1.77–4.44	
Divorced		1.17	0.48–2.86		1.26	0.54–2.97	
**Catholic**				0.002			0,026
No		Ref.			Ref.		
Yes		0.50	0.32–0.77		0,58	0,37–0,90	
**Evangelical**				0.012			0.867
No		Ref.			Ref.		
Yes		1.62	1.11–2.37		1,04	0.66–1.65	
**Family and life experiences about violence**							
**Maternal aggression by intimate partner**				<0.001			0.004
No		Ref.			Ref.		
Yes		2.31	1.58–3.38		1.78	1.21–2.61	
**Sexual violence in childhood**				0.003			0.088
No		Ref.			Ref.		
Yes		2.03	1.28–3.22		1.52	0.94–2.47	
**Behavioral variables**							
**Average frequency of alcohol use**				0.036			0.456
Never		Ref.			Ref.		
Monthly or less		0.73	0.43–1.24		0.67	0.40–1.12	
2–4 times per month		0.97	0.57–1.66		0.94	0.51–1.72	
2–3 times per week		1.17	0.62–2.22		0.86	0.45–1.63	
≥4 times per week		3.31	1.40–7.79		1.34	0.60–2.96	
**Smoking**				0.002			0.304
Never smoked		Ref.			Ref.		
Current smoker		2.25	1.44–3.51		1.39	0.87–2.22	
Former smoker		1.39	0.81–2.37		1.30	0.76–2.21	

N: absolute frequency; 95%CI: 95% confidence interval; PR: prevalence ratio; Ref.: reference group.

Sexual violence was 97% more prevalent among non-white women than in self-reported white women (PR: 1.97; 95%CI 1.10–3.53, p<0.05). Among those with up to eight years of schooling, a 138% higher prevalence was observed compared to those with 12 years or more (PR: 2.38; 95%CI 1.31–4.33). Women whose mothers had a history of intimate partner violence had about 2.4 times more frequency of sexual violence, and those who consumed alcohol during four days or more had a 192% higher prevalence of sexual violence compared to those who never consumed alcohol (PR: 2.92; 95%CI 1.59–5.36, p<0.01) (Table 4).

**Table 4:** Crude and adjusted analysis of the prevalence of sexual violence in the last 24 months according to sociodemographic characteristics, family and life and behavioral experiences among women living in an urban area in the municipality of Vitória, Espírito Santo, Brazil, 2022 (N = 1,086).

Characteristics	N	Crude analysis			Adjusted analysis		
		PR	95%CI	p-value	PR	95%CI	p-value
**Sociodemographic**							
**Age group (years)**				0.162			0.269
18 to 29		2.59	1.19–5.65		2.46	0.96–6.34	
30 to 39		1.86	0.81–4.26		2.20	0.86–5.66	
40 to 49		2.36	1.08–5.15		2.80	1.15–6.82	
50 to 59		1.93	0.84–4.43		2.10	0.85–5.18	
60 or more		Ref.			Ref.		
**Self-declared skin color**				0.003			0.022
White		Ref.			Ref.		
Non-white		2.31	1.34–3.97		1.97	1.10–3.53	
**Complete years of schooling**				<0.001			0.017
0 to 8		3.14	1.77–5.56		2.38	1.31–4.33	
9 to 11		2.04	1.16–3.57		1.60	0.89–2.86	
≥12		Ref.			Ref.		
**Family income in tertiles**				0.005			0.675
1st: poorest		2.45	1.33–5.52		0.80	0.37–1.73	
2^nd^		1.32	0.66–2.63		1.01	0.44–2.31	
3rd: richest		Ref.			Ref.		
**Marital status**				0.017			0.072
Married		Ref.			Ref.		
Consensual union		1.22	0.70–2.15		1.08	0.60–1.94	
Single		2.46	1.40–4.31		2.08	1.12–3.89	
Divorced		1.47	0.59–3.65		1.64	0.66–4.07	
**Catholic**				0.054			0.806
No		Ref.			Ref.		
Yes		0.62	0.38–1.01		0.93	0.54–1.62	
**Evangelical**				0.089			0.963
No		Ref.			Ref.		
Yes		1.48	0.94–2.33		0.99	0.56–1.75	
**Family and life experiences about violence**							
**Maternal aggression by intimate partner**				<0.001			<0.001
No		Ref.			Ref.		
Yes		2.86	1.81–4.52		2.36	1.51–3.69	
**Sexual violence in childhood**				0.010			0.117
No		Ref.			Ref.		
Yes		2.07	1.19–3.60		1.60	0.89–2.88	
**Behavioral variables**							
**Average frequency of alcohol use**				0.006			0.006
Never		Ref.			Ref.		
Monthly or less		0.90	0.49–1.65		0.85	0.46–1.60	
2–4 times per month		1.00	0.52–1,94		1.26	0.65–2.45	
2–3 times per week		1.40	0.67–2,93		1.55	0.72–3.34	
≥4 times per week		4.96	2.06–11,94		2.92	1.59–5.36	
**Smoking**				0.039			0.327
Never smoked		Ref.			Ref.		
Current smoker		2.00	1.15–3.49		1.35	0.73–2.50	
Former smoker		1.51	0.82–2.79		1.57	0.82–3.00	

N: absolute frequency; 95%CI: 95% confidence interval; PR: prevalence ratio; Ref.: reference group.

## Discussion

We observed that the prevalence of violence against women by an intimate partner (psychological, physical, or sexual) in the previous 24 months, i.e., during the pandemic. The psychological violence was the most frequent, with a 20.2% prevalence, followed by physical violence. Sexual violence in the previous 24 months had the lowest prevalence.

Ethiopia presents data similar to those found in this study, showing a 24.6% prevalence of intimate partner violence. Psychological violence was the most frequent [29]. In Jordan and Lebanon, higher values were found: 40% and 40.7% [30, 31], also showing psychological violence as the most commonly perpetrated by the intimate partner and associating being married and not having a source of income or employment [31]. Other studies also reinforce the significant increase in violence against women during the pandemic period, especially psychological and physical violence [14, 29].

Evaluating the dimension of this increase, an Italian study found that over 40% of the interviewed victims reported an increase in violent attitudes by their partner during confinement, with emphasis on psychological violence (54.5%), threats (44.1%), physical violence (43.4%), economic violence (46.7%) and children witnessing violence against their mother (44.8%) [8].

In this context, women’s vulnerability increases as men use violence as an expression of anger or shame when they do not reach hegemonic male ideals [31].

During the COVID-19 pandemic, domestic coexistence, excessive alcohol consumption, and, consequently, men’s control over women increased due to the many economic and emotional instabilities experienced. These women living with the aggressor were also restricted from free access to support institutions, which, in turn, had their reception capacity decreased, such as shelters and hotels, and difficulty in accessing health services adapted to pandemic control measures, canceling elective care. In many cases, the woman being confined indoors with a violent partner proved to be more dangerous than the COVID-19 contamination itself during the pandemic [29].

The association between lower schooling levels and higher prevalence of violence corroborates another study [27]. The qualification of women by years of study may lead to lower tolerance to violence and closer approach to ways of coping with it. Higher education is directly related to greater financial autonomy, which, despite being a facilitator for women to leave violent relationships, also increases the risk of more severe victimization [32, 33]. Women with higher educational levels would have more resources to achieve greater autonomy and could have more skills to recognize and break away from abusive relationships [32].

Also regarding income, women belonging to the second tertile of family income had 62% more occurrence of psychological violence compared to those of the third tertile (higher income). Economic factors, such as lower income, are associated with a higher occurrence of intimate partner violence [34]. The scarcity of financial resources creates a limited access to basic need products, such as food and hygiene, causing additional stress in the relationship with the partner and, thus, favoring the occurrence of situations of partner violence against women [35, 36].

Regarding marital status, we observed a higher frequency of psychological violence (83%) and physical violence (180%) among single women compared to married women. Other studies corroborate these results [37, 38]. This data may reinforce the former intimate partner’s non-acceptance of the end of the relationship and, by knowing the place of residence and the social and/or professional life of the victim, persecute and coerce woman, leading to violence [39].

Regarding religion, women who referred to themselves as catholic showed a lower prevalence of psychological (-25%) and physical (-42%) violence compared to non-Catholic women. Religious groups can provide a sense of belonging, promoting the socialization and inclusion of women in a new moral community. In this environment, women share social assets that help and protect them [40]. Another point is that religion can influence how gender violence is dealt with, since the roles of submission attributed to women within religious institutions can contribute to the non-recognition of violent attitudes and difficulty in breaking the cycle of violence [41].

Our study showed that women whose mothers had been victims of intimate partner violence had a 34% higher prevalence of psychological, physical, and sexual violence. A Bangladeshi study found that the participating women who witnessed their mothers suffer physical violence throughout their lives had an association with a higher prevalence of intimate partner violence in adulthood [42]. Another study that investigated if exposure to parents’ intimate partner violence during adolescence led to increased involvement in IPV during adulthood showed that this exposure resulted in an increased risk of violence later [24]. It is possible that from an early age individuals become tolerant of violence against women and start to consider it as an acceptable action that permeates marital relations, as well as a possible form of conflict resolution [42].

Another point about life experience identified in this study was that women who suffered sexual violence during childhood had a higher prevalence of psychological and physical abuse by their intimate partner. This finding corroborates a study that showed that women who suffered sexual violence during childhood were more likely to be victims of physical violence by an intimate partner during adulthood compared to those who did not [43]. A systematic review with meta-analysis also showed that all types of child abuse, which includes physical, psychological, and sexual abuse, as well as neglect, were related to the higher occurrence of intimate partner violence during adulthood [44].

Violence during childhood has several negative effects on the individual’s life, such as unsafe and disorganized attachment [45], low self-esteem, low quality of relationships [46], and naturalization of violent relationships [47]. Thus, it is possible that in adulthood these factors contribute to such people becoming more vulnerable to being in violent relationships. However, it is important to emphasize that having been a victim of childhood violence is not the only associated or predictive factor for violence in adulthood, and other factors should be considered.

Sexual violence was 97% more prevalent among non-white women than in self-reported white women. Other studies have found higher frequencies of sexual violence among non-white women; however, with no significance after adjustments [48, 49]. Race should be considered a social variable, historically and culturally constructed, and that suggests a determinant of the lack of equity in health among racial groups. Thus, being non-white, especially Black, functions as a marker of social disadvantage, and this factor behaves as an intermediary for unfavorable socioeconomic situations [50]. Therefore, this population can accumulate vulnerabilities, hindering the break up process with the aggressor and chronic situations of violence [51].

Regarding behavioral characteristics, for alcohol consumption for four days or more, the occurrence of sexual violence was 192% higher compared to those who never consumed alcohol. In a systematic review study that analyzed the association between alcohol use by women and experience of victimization of physical and/or sexual violence by an intimate partner, a positive association between alcohol use and intimate partner victimization among women was observed [52].

A study conducted in the United States showed that women with a recent history of intimate partner violence were more likely to consume more alcohol compared to women who did not suffer abuse [53]. The literature suggests that alcohol consumption is a contributing cause, or a moderating factor that increases aggressive behavior in severity, since consuming it does not result in aggression in all individuals and in all circumstances [54]. Data collection in our study was performed during the pandemic; thus, it is worth noting that the literature shows a higher alcohol use during this period, which may have further exacerbated the situation of domestic violence [55].

The study of violence still encompasses certain difficulties in the process of defining and accepting the act by the victim, especially when the aggressors are people close to them. One of this study’s limitations is the information bias considering the many questions related to the concept of violence that can influence the accuracy of information provided by the participants. This can lead to an underestimation of the prevalence presented; however, to minimize it, we highlight that the interview was always conducted in a private space, where only the interviewee and interviewer were present. Moreover, reverse causality can occur due to the cross-sectional design of the study, considering that some of the variables measured cannot determine their temporality, especially regarding behaviors, such as alcohol use, religion, and marital status. However, they are important variables for public health in the context of violence and should be measured. In this study, the association between violence was not presented, such as the occurrence of different types of violence intimate partner. Therefore, it is recommended that other studies be carried out to analyze the co-occurrence of types of violence. Thus, despite the limitations, this study shows alarming data on violence against women during the pandemic and draws attention to the vulnerability of this group in the domestic environment, which should be a place of rights protection.

## Conclusion

The prevalence of violence psychological against women perpetrated by an intimate partner during the pandemic was the most frequent, followed by physical and sexual violence. This study identified associations of violence against women during the pandemic perpetrated by their partners with sociodemographic characteristics, family and life experiences about violence, and behavioral characteristics.

It is necessary to be aware of this profile in victims of violence against women perpetrated by their intimate partner during a pandemic period, considering that this is a moment in which several strategies are conducted to contain the disease’s spread, such as social isolation, which can worsen the scenario of gender violence. Since certain characteristics are associated with a higher prevalence of these types of violence, it is expected that the data discussed in this study help to create and plan public policies to combat gender violence perpetrated by intimate partners.
